# proNGF Measurement in Cerebrospinal Fluid Samples of a Large Cohort of Living Patients With Alzheimer's Disease by a New Automated Immunoassay

**DOI:** 10.3389/fnagi.2021.741414

**Published:** 2021-10-27

**Authors:** Francesca Malerba, Ivan Arisi, Rita Florio, Chiara Zecca, Maria Teresa Dell'Abate, Bruno Bruni Ercole, Serena Camerini, Marialuisa Casella, Giancarlo Logroscino, Antonino Cattaneo

**Affiliations:** ^1^Fondazione EBRI (European Brain Research Institute) Rita Levi-Montalcini, Rome, Italy; ^2^Institute of Translational Pharmacology - National Research Council (IFT-CNR), Rome, Italy; ^3^Center for Neurodegenerative Diseases and the Aging Brain, Department of Clinical Research in Neurology of the University of Bari “Aldo Moro” at “Pia Fondazione Card. G. Panico” Hospital Tricase, Lecce, Italy; ^4^Core Facilities, Istituto Superiore di Sanità, Rome, Italy; ^5^Department of Basic Medicine Sciences, Neuroscience, and Sense Organs, University of Bari “Aldo Moro”, Bari, Italy; ^6^BIO@SNS Laboratory, Scuola Normale Superiore, Pisa, Italy

**Keywords:** Alzheimer's disease, proNGF, immunoassay, biomarker, diagnosis, neurodegenerative diseases

## Abstract

The discovery of new biomarkers for Alzheimer's disease (AD) is essential for an accurate diagnosis, to conceive new strategies of treatments, and for monitoring the efficacy of potential disease-modifying therapies in clinical trials. proNGF levels in the cerebrospinal fluid (CSF) represent a promising diagnostic biomarker for AD, but its validation was hampered by the absence of a reliable immunoassay. In the literature, proNGF is currently measured in postmortem brain tissue by semiquantitative immunoblot. Here we describe the development and validation of a new method to measure proNGF in the CSF of living patients. This method, based on molecular size separation by capillary electrophoresis, is automated and shows a 40-fold increase in sensitivity with respect to the proNGF immunoblot, largely used in literature, and is robust, specific, and scalable to high-throughput. We have measured proNGF in the cerebrospinal fluid of 84 living patients with AD, 13 controls, and 15 subjective memory complaints (SMC) subjects. By comparing the proNGF levels in the three groups, we found a very significant difference between proNGF levels in AD samples compared with both controls and SMC subjects, while no significant difference was found between SMC and controls. Because of the development of this new immunoassay, we are ready to explore the potentiality of proNGF as a new biomarker for AD or subgroups thereof, as well as for other neurodegenerative diseases.

## Introduction

Alzheimer's disease (AD) is characterized by a long preclinical stage and a clinical stage of variable duration that precedes the dementia stage (Jack et al., 2010; Dubois et al., 2016). Indeed, when patients with AD are first diagnosed as demented, even if in the mild stage, 70% of cells in critical areas like the hippocampus have been already damaged or dead.

The central diagnostic role of imaging and cerebrospinal fluid (CSF) biomarkers have been confirmed in the revised NIA-AA diagnostic criteria (Jack et al., 2018). To date, no single biomarker is diagnostic, and the combination of different biomarker data and techniques is necessary.

The discovery of new CSF biomarkers targeting early phases of AD would be essential for an accurate diagnosis, to conceive new strategies of treatments, and for monitoring the efficacy of potential disease-modifying therapies in clinical trials.

proNGF, the nerve growth factor (NGF) precursor, represents a good candidate to become a new CSF biomarker for AD. Indeed, it was demonstrated that an increase in proNGF level was associated with neurodegeneration in early AD and that proNGF/NGF ratio is an upstream driver for neurodegeneration both in animal models and in humans (Capsoni et al., 2000, 2010; Counts and Mufson, 2005; Capsoni and Cattaneo, 2006; Tiveron et al., 2013; Iulita and Cuello, 2014; Counts et al., 2016; Fasulo et al., 2017).

However, the clinical validation of proNGF as a candidate biomarker for AD and other neurodegenerative conditions has been hampered by the lack of a reliable immunoassay. So far, a proNGF immunoassay able to detect proNGF in biological fluids is not yet available. Some commercial proNGF ELISA kits exist, but they are only validated for cell supernatant or cell lysates from proNGF-transfected cells. Moreover, we recently demonstrated that NGF and proNGF reciprocally interfere with the experimental outcome, making the measurements of both NGF and proNGF unreliable and dependent on the unknown proNGF/NGF ratio (Malerba et al., 2016). Currently, despite the often frequent inappropriate use of proNGF ELISA, the only reliable way to measure NGF and proNGF, without such reciprocal interferences, is the semiquantitative immunoblot, exploiting their difference in molecular mass (Tiveron et al., 2013; Counts et al., 2016; Pentz et al., 2020). However, the sensitivity and the throughput of immunoblot are low.

Here we describe a new method to measure proNGF, in the absence of interference by mature NGF, and have applied the method to the determination of proNGF in the CSF of living patients. This new immunoassay, based on capillary electrophoresis, exploits the differences in molecular weight (MW) between NGF and proNGF in order to separate the two forms, avoids the severe limitation of the reciprocal interference (Malerba et al., 2016), is automated, and has a greater sensitivity than the Western blot. The results on the proNGF levels in the CSF from living patients provide the largest dataset so far and offer a reliable method toward the use of proNGF levels as a biomarker in the Alzheimer's continuum, to stratify patients and to distinguish different neuropathological entities that may be confounded with AD.

## Materials and Methods

### Patients

A total of 112 participants (84 patients with AD, 15 with SMC, and 13 control subjects), referred to the Center for Neurodegenerative Diseases and the Aging Brain of the University of Study of Bari “Aldo Moro” at Pia Fondazione “Card. Panico” Hospital (Tricase), were enrolled in this study. Each patient underwent a multidisciplinary assessment with a neurological and neuropsychological examination, an MRI-3T scan, a routine laboratory assessment, and a lumbar puncture for CSF biomarkers analysis, as part of the diagnostic procedure. All study participants gave their written informed consent, and the study was approved by the Local Ethical Committee, according to the Declaration of Helsinki. The detailed medical examination, the inclusion criteria, and the CSF biomarkers analysis undergone by these patients are reported in the Supplementary Material.

### proNGF Immunoassay

The calibration curve was set up by carrying out serial dilutions of human recombinant proNGF produced in our lab (Malerba et al., 2015), from 4 μg/ml to 31 ng/ml in 0.1% Wes Sample Buffer (Biotechne, Minneapolis, MN, USA). The proNGF dilutions were added with one-fifth of Fluorescent Master Mix (Biotechne) and then boiled. The area under the peak is proportional to the proNGF concentration. The experimental points were interpolated by a second-order polynomial function, using GraphPad Prism.

The CSF (130 μl) was desalted by Zeba Spin Desalting Columns (7K MWCO Thermo Scientific, Waltham, Massachusetts, USA), following the instructions of the manufacturer. The proteins in the sample were precipitated with 20% TCA. The protein pellet was resuspended in 10 μl of 0.1% Wes Sample Buffer, added with one-fifth of Fluorescent Master Mix (Biotechne), and then boiled. 130 μl of CSF is enough to carry out six replicates.

Where not differently specified, the steps of the run followed the standard protocol of the manufacturer.

The calibration curve and samples were run in duplicates on Simple Wes (Biotechne) by using 2–40 kDa cartridges. Anti-NGF MyBiosource, San Diego, CA, USA (MBS125020) and the Biotin-SP AffiniPure Goat Anti-Rabbit IgG (111-065-003 Jackson Immunoresearch, Cambrideshire, UK) were used as primary and secondary antibodies, respectively.

After the run, data are displayed by an electropherogram, in which quantitative results such as MW, signal intensity (area of the peak), % area, and signal-to-noise ratio for each immunodetected protein are calculated and listed. The peaks are so identified by MW, and the area under the peak is proportional to the protein concentration. For each peak, a signal-to-noise ratio of ≥10 was considered acceptable. Quantitative analysis was carried out using the software Compass, specific for Simple Wes.

The immunoassay was validated first with artificial CSF spiked with human recombinant proNGF and then with eight CSF samples from patients. We also checked the cross-reactivity, the specificity of the assay, the effect of sample processing on the reliability, and the inter- and intra-assay robustness. We also measured the increase in sensitivity with respect to a proNGF Western blot. Moreover, we identified the proNGF peaks by two different experiments: an immunodepletion experiment and a mass spectrometry (MS) analysis. The details of the methods of all these steps of validation and analysis are described in the Supplementary Material.

### Clinical Sample Measurement

Using the proNGF immunoassay, 84 patients with AD, 15 SMC subjects, and 13 controls were measured. Each sample was tested at least four times, in two different assays. For each sample, the mean, SD, and coefficient of variation (CV) were calculated. A CV of ≤ 20% was considered acceptable. In the case of CV > 20%, the measurements were repeated. For each peak, the area under the peak was computed by the supplied software Compass (Biotechne) and analyzed statistically.

### Statistical Analysis

Kruskal–Wallis test was used for single-factor analysis to compare the biomarker levels between the three diagnostic groups (AD, SMC, and controls). Pairwise Mann–Whitney test, with multiple testing *p*-value corrections, was used as a *post-hoc* test. Correlation between biomarkers was quantified by Spearman's index. Diagnostic performance of biomarkers was analyzed by logistic regression models and ROC curves: the response variable is the binarized diagnosis (AD = 1 vs. SMC_or_Control = 0), while predictors are the standardized biomarker measures, that is, biomarker_std = (biomarker— <biomarker>)/SD, with biomarker = Aβ42/Tau/pTau/proNGF, <biomarker> = average value, SD = standard deviation. The ROC curves were compared by the one-sided De Long Z-test. All statistical analyses were performed using R-Bioconductor, including pROC and ggplot2 packages (Robin et al., 2011; Team, 2013; Villanueva et al., 2016).

## Results

### proNGF Immunoassay: Development and Validation

To set up a novel proNGF immunoassay, we exploited capillary electrophoresis performed with Simple Wes (ProteinSimple, San Jose, CA, USA), an instrument that integrates and automates the entire protein separation and detection processes. Protein separation by a capillary electrophoresis platform is followed by the detection of the protein of interest with primary, secondary, and tertiary antibodies and standard chemiluminescent signals. Sample data are displayed by an electropherogram, in which quantitative results such as MW, signal intensity (area of the peak), % area, and signal-to-noise ratio for each immunodetected protein are calculated and listed.

**Figure 1:**
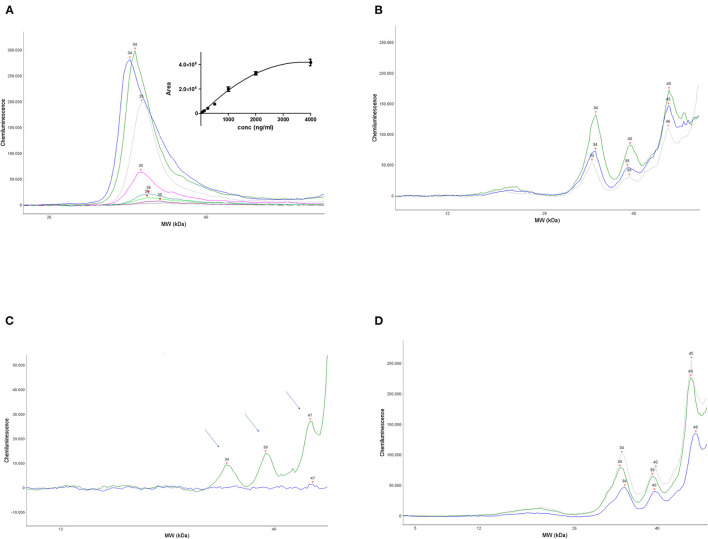
Set up and validation of the immunoassay. **(A)** A representative calibration curve obtained by serial dilutions of human recombinant proNGF. Main panel: overlapped electropherograms of the different concentrations of proNGF from Simple Wes runs. Inset panel: interpolation by a polynomial curve of the values of the area under the peaks for each proNGF concentration. Dynamic range (4,000–31 ng/ml). **(B)** Three representative electropherograms of CSF samples from three different patients with AD. **(C)** Electropherograms obtained from the immunodepletion experiment. A CSF sample from the same patient was divided into two aliquots: one aliquot was processed normally and the other one was immunoprecipitated by αD11 and then processed. The immunodeprived sample (blue electropherogram) and the normal processed sample (green electropherogram) were run side by side on Simple WES. The arrows indicate the three peaks that disappear in the immunodeprived sample. **(D)** Three representative electropherograms of CSF samples from an AD patient (blue), an SMC subject (gray), and a control subject (green).

Figure 1A shows a representative electropherogram obtained by overlapping the plots corresponding to the calibration curve, run with different amounts of human recombinant proNGF. The proNGF peaks correspond to an apparent MW of about 34 kDa, based on the Simple Wes standards. Recombinant proNGF from *Escherichia coli* has a theoretical MW of 25 kDa, but due to its strong cationic charge, it runs with an apparent MW >30 kDa, also in SDS PAGE. The Simple Wes run is influenced by many factors: the pH of the running buffer as well as the nature of the stacking and the separation matrix or gel, as well as the sample concentration, which determine the observed slight peak shifts between the different points of the calibration curve. As such, the Simple Wes does not provide an absolute MW but an observed one (manufacturer instructions).

The area under the peak is proportional to the proNGF concentration in that sample. The interpolated calibration curve shown in the inset of Figure 1A, obtained from 12 independent experiments, shows that the dynamic range of the assay is between 31 ng/ml (corresponding to 124 pg in the sample) and 2,000 ng/ml.

As described in the “Materials and methods” section, the assay was also validated with eight CSF samples from patients, where a proNGF peak was found to be well-detected, at the same MW of the recombinant proNGF, and its concentration was estimated by comparison with the calibration curve.

In Figure 1B, the electropherograms of three representative CSF samples from three patients with AD are shown. In all eight human CSF samples analyzed at this pilot stage, a prominent peak corresponding to that of the recombinant proNGF is evident. In addition, in some samples, immunoreactive peaks corresponding to lower MWs in the nominal range of 16–23 kDa are visible, corresponding to mature NGF (see below). Remarkable is also the presence, in almost all the CSF samples tested, of larger NGF immunoreactive bands of higher MW (one peak of about 39 kDa and another one in the nominal range of 45–50 kDa). These higher MW peaks are not present in the samples of artificial CSF spiked with recombinant proNGF and represent, therefore, forms of proNGF naturally produced in the brain, most likely by posttranslational modifications, as discussed below.

The same samples were run with and without the primary or the secondary antibody. Moreover, Simple Wes Fluorescent Master MIx were run in the absence of a CSF sample, in order to check for a possible cross-reactivity by the antibodies. As evident from Supplementary Figure 1, in all the cases, there is no cross-reactivity between the reagents in the MW range of interest. As evident from the comparison of the pherograms, the peak at 2 kDa is generated by the non-specific interaction between the Fluorescent Master Mix and the anti-rabbit biotinylated antibody, but it does not compromise the analysis.

To check the specificity of the assay, an immunodepletion of a CSF sample was carried out. A CSF sample from the same patient was divided into two aliquots: one was processed normally, while the other was immunoprecipitated by the anti-NGF MAb αD11 (Cattaneo et al., 1988), the monoclonal antibody able to recognize a specific epitope of the mature NGF and to immunoprecipitate both NGF and proNGF (Tiveron et al., 2013). After processing, the samples were run side by side on Simple Wes. Figure 1C shows that the peak corresponding to the MW of 34, 39, and 47 kDa disappeared in the immunodepleted CSF sample (blue line), allowing us to conclude that they are specifically recognized by anti-NGF Mab αD11.

The 34 kDa peak in the CSF samples corresponds to the MW of the recombinant proNGF (as in Figure 1A), purified from *E. coli*, and corresponds, therefore, to the naked proNGF protein, without any posttranslational modification. The immunodepletion experiments allowed to demonstrate the identity of two other specific peaks, with an apparent MW of 39 and 47 kDa (actually in the range of 45–50 kDa, in almost all the samples). We can hypothesize that these higher MW peaks may be proNGF species holding posttranslational modifications. Higher MW forms of proNGF, which result also from protein modifications due to oxidative damage, have been detected by Western blot in the human brain and the CSF of patients with AD (Pedraza et al., 2005; Kichev et al., 2009). Also, other authors reported proNGF forms at an MW of about 40 and 50 kDa in immunoblot (Lakshmanan et al., 1988; Reinshagen et al., 2000; Bruno and Cuello, 2006; Pentz et al., 2020).

As reported in the “Materials and methods” section, the CSF samples were processed and concentrated (13:1) by TCA. To exclude any effect of the TCA precipitation step on the results, we choose three samples with a large amount of proNGF and tested them neatly. The samples were run side by side, neat, and concentrated. The areas under the 34, 39, and 45–50 kDa peaks were compared in the two sets of measurements, after normalization of the TCA set by the concentration ratio (13), by the one-sample two-sided *t*-test. The measurements corresponding to the 34 kDa peak gave consistent results between the TCA concentrated and the neat samples: the actual ratio was 12.600, with a confidence interval of the difference from 1.450 to 0.650, and the two-tailed *p*-value was 0.4401, thus largely not statistically significant. The data for the larger MW peaks (39 and 45–50 kDa) gave, instead, inconsistent results, with respect to the dilution ratio (data not shown). For this reason, we decided to carry out the statistical analyses of proNGF levels in CSF only on the 34 kDa peak, corresponding to unmodified proNGF. The reasons for the inconsistent results with the two larger bands remain to be investigated.

### Comparative Sensitivity of the proNGF Immunoassay

To establish the sensitivity of our assay, the detection limits for recombinant human proNGF in Simple Wes and Western blot were compared by loading decreasing amounts of human recombinant proNGF (from 800 to 6.2 ng per lane). The membrane was challenged by the same antibody used in Simple Wes (anti-NGF MBS125020 MyBiosource, a method described in Supplementary Material). The lowest dilution detected was 25 ng/lane, corresponding to a concentration of 1,250 ng/ml in the sample (Supplementary Figure 2). Since Simple Wes could detect 31 ng/ml of proNGF (Figure 1A), the increase in sensitivity of the new proNGF immunoassay, with respect to the standard, currently used Western blot, is 40-fold.

Is this difference in sensitivity between the two immunoassays dependent on the particular antibody used for detection? In principle, antibodies exhibiting good performance in Simple Wes might not work so well in the Western blot and vice versa. In Western blot experiments, previously carried out in the lab with the anti-NGF mAb H20 from Santa Cruz Biotechnology, Dallas, Texas, USA (sc-548), we were able to detect a lower limit of 5 ng/lane of human recombinant proNGF, corresponding to a concentration of 250 ng/ml in the sample. Since by Simple Wes we could detect 31 ng/ml of proNGF with the anti-NGF MBS125020, the increase in sensitivity is about 8-fold. Unfortunately, the anti-NGF PAb H20 is no longer commercially available, so the quantitative side-by-side comparison of the two immunoassays with that other antibody cannot be performed.

We conclude that the sensitivity of the Simple Wes proNGF immunoassay is 40-fold higher than that of a Western blot immunoassay, using the same, well-validated antibody.

### Identification of proNGF in the 34 kDa Peak by Mass Spectrometry

To directly demonstrate the presence of proNGF in the peaks found in Simple Wes from CSF samples, an MS experiment was carried out. CSF samples from eight patients were pooled, immunoprecipitated with anti-NGF mAb αD11, and run on SDS-PAGE with adequate controls (see Supplementary Figure 3). On lane 5, corresponding to the immunoprecipitated CSF pool, three bands at the MW of about 50, 30, and 25 kDa, indicated in the Supplementary Figure S3, as 1, 2, 3, were identified, purified and analyzed by MS. The identity of these bands, as containing proNGF forms, was confirmed by Western blot analysis, challenged with the anti-proNGF ANT005 (Alomone, Jerusalem, Israel) and the anti-NGF MBS125020 (MyBiosource), as described in the Supplementary Material. As evident from Supplementary Figure 4, on lane 2, corresponding to the immunoprecipitated CSF pool, the anti-proNGF ANT005 detected the same pattern of proNGF bands of the SDS-PAGE, while MBS125020 is less efficient, detecting two out of three of the proNGF bands (30 and 50 kDa), despite the long exposition time. This is expected due to MBS125020 low sensitivity in the Western blot, as previously discussed.

The MW of the bands identified by SDS-PAGE did not correspond to the MW of the Simple Wes peaks. As previously mentioned, the two techniques are different and both give only an apparent MW for each protein. However, since the recombinant proNGF was run side by side both in Simple Wes and in SDS-PAGE, we can certainly conclude that band no. 2 in the SDS PAGE at 30 kDa (Supplementary Figure 3, compare lanes 3,4 with lane 5) corresponds to the peak of apparent MW 34 kDa in Simple Wes (Figure 1B).

The MS analysis identified, in each of the three bands, the protein beta-nerve growth factor (Swiss Prot accession P01138), with 5 (in band 1) or 6 (in bands 2 and 3) unique peptides. As an example, the MS/MS spectra of beta-nerve growth factor peptides identified in band 2 are shown in Supplementary Figure 5. The experiment was carried out three times on three independent CSF pools.

### Determination of proNGF Levels in Human CSF Samples

Having validated a sensitive and robust proNGF immunoassay, we used it to analyze the CSF samples from 84 patients with AD, 15 SMC subjects, and 13 controls (Table 1).

**Table 1:** Sociodemographic and biomarker details of the study population.

	**CTR**	**SMC**	**AD**
*n* (Male + Female)	5 + 8	6 + 9	28 + 56
Age [years] (mean ± SD)	65.2 ± 5.2	54.6 ± 10.8	68.8 ± 7.1
Ab42 [pg/mL] (mean ± SD)	934.9 ± 286.8	1,000.5 ± 184.5	485.4 ± 142.0
Tau [pg/mL] (mean ± SD)	165.2 ± 80.0	230.2 ± 71.9	647.3 ± 339.8
pTau [pg/mL] (mean ± SD)	32.8 ± 11.5	39.7 ± 11.2	86.2 ± 51.7
MMSE (mean ± SD)	n.a.	n.a.	17.1 ± 5.4

The areas corresponding to the proNGF of the apparent MW of 34 kDa were then analyzed. In Figure 1D, three representative samples from the different diagnosis groups are shown. In Table 2, the concentrations of proNGF, obtained by interpolating on the calibration curve the median and the average values of the areas of each analyzed group, are listed.

**Table 2:** Mean and median of proNGF peaks area and proNGF concentration for the three diagnostic groups.

	**Diagnosis**	**Area**	**Coefficient of variation (%)**	**Concentration (ng/ml)**	**Concentration (nM)**
proNGF mean	AD	990,902	11.0	502.9	20.1
	CTR	1,475,921	11.2	759.4	30.4
	SMC	1,554,784	9.5	803.3	32.1
proNGF median	AD	960,578	10.9	487.5	19.5
	CTR	1,404,631	9.6	720.4	28.8
	SMC	1,458,425	6.4	750	30.0

**Figure 2:**
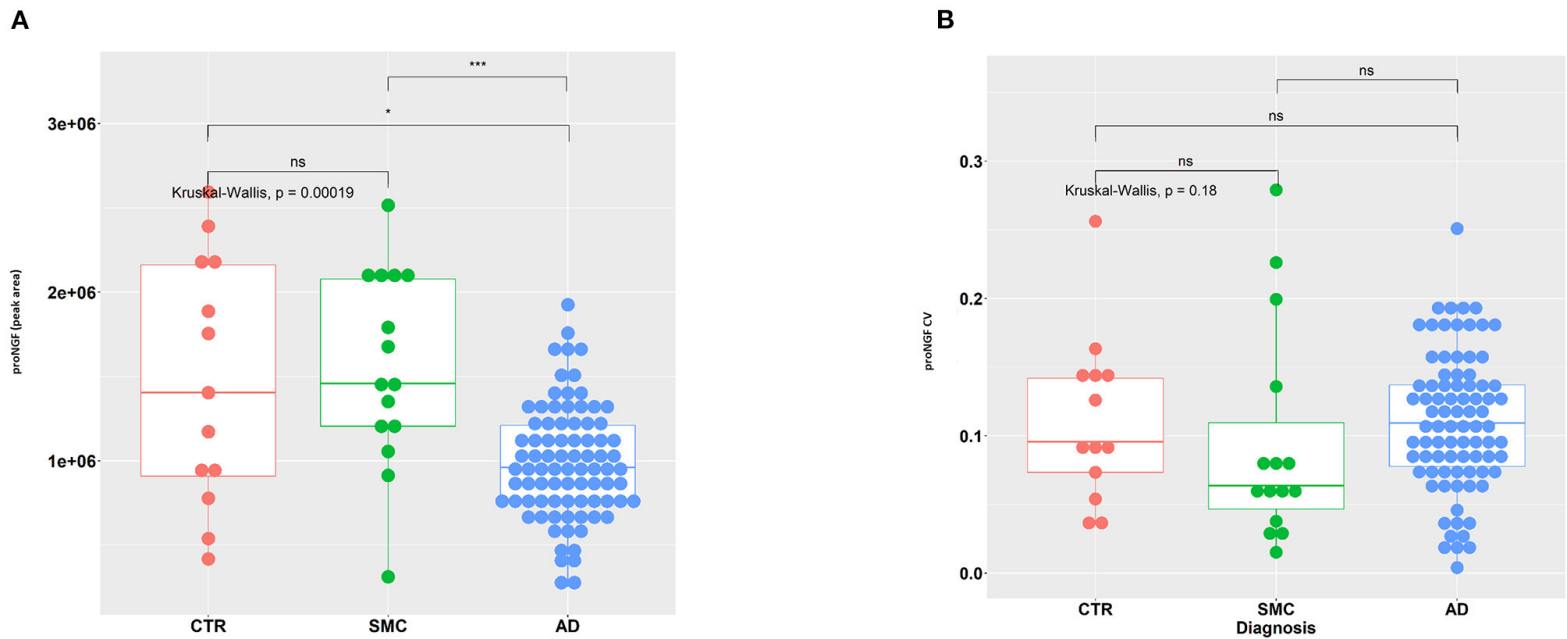
Statistical analysis of proNGF in the CSF samples. **(A)** A measure of proNGF (peak area) in the three diagnostic groups. Kruskal–Wallis test is followed by pairwise Mann–Whitney test with *p*-value correction (horizontal bars). The difference in data dispersion between groups was analyzed by the variance *F*-test. **(B)** Coefficient of variation (SD/mean) for proNGF measures in the three diagnostic groups. Kruskal–Wallis test is followed by pairwise Mann–Whitney test with *p*-value correction (horizontal bars). (*)*p* < 0.05, (***)*p* < 0.0001, for *post-hoc* test.

In Figure 2A, the area of the proNGF 34 kDa peak for all samples is reported: there is a very significant difference between the AD group, with respect to both SMC and control groups. We can also observe a clearly larger dispersion of data in SMC and control groups compared with the AD group (Variance *F*-test, *p* = 1.15E-05 and *p* = 1.91E-03 for AD vs. SMC and AD vs. Control, respectively), while no significant difference in variance exists between SMC and control groups. The coefficient of variation is largely below 20% for almost all the samples, with an average value around 10% (Figure 2B), thus confirming a low technical variability of the immunoassay.

The difference in the level of the 34 kDa proNGF between the AD samples with respect to the SMC and control groups is very interesting but does not allow yet a differential diagnosis for patients with AD. This will require the comparison of AD samples with samples of other neurodegenerative diseases (work in progress).

### Correlation of proNGF Levels With the Alzheimer's Biomarkers

**Figure 3:**
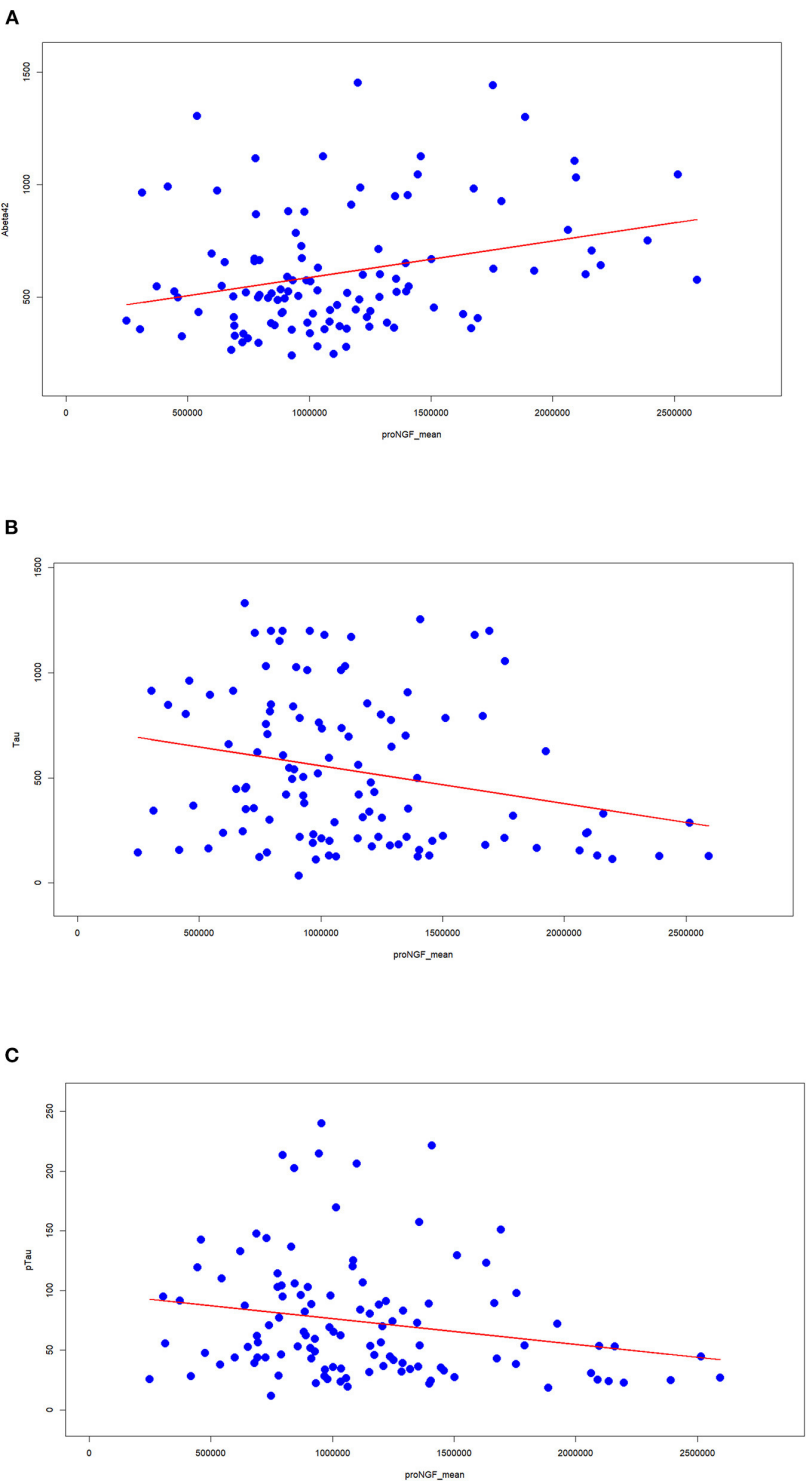
Correlation between proNGF and the clinically validated biomarkers. The Spearman's index and the corresponding statistical significance are computed between proNGF levels (peak area) and the three classical AD biomarkers [**(A)** Aβ42; **(B)** tau; **(C)** pTau].

We assessed the classical AD biomarkers in the set of CSF samples. The measured proNGF levels (34 kDa peak) are significantly correlated with the major assessed AD biomarkers. In particular, proNGF levels are directly correlated with Aβ42 (Figure 3A) and inversely with Tau (Figure 3B) and pTau (Figure 3C). The low correlation index, though being significant, suggests that proNGF may provide a partial alternative biological information compared with the other biomarkers and, at the same time, a further diagnostic accuracy.

**Figure 4:**
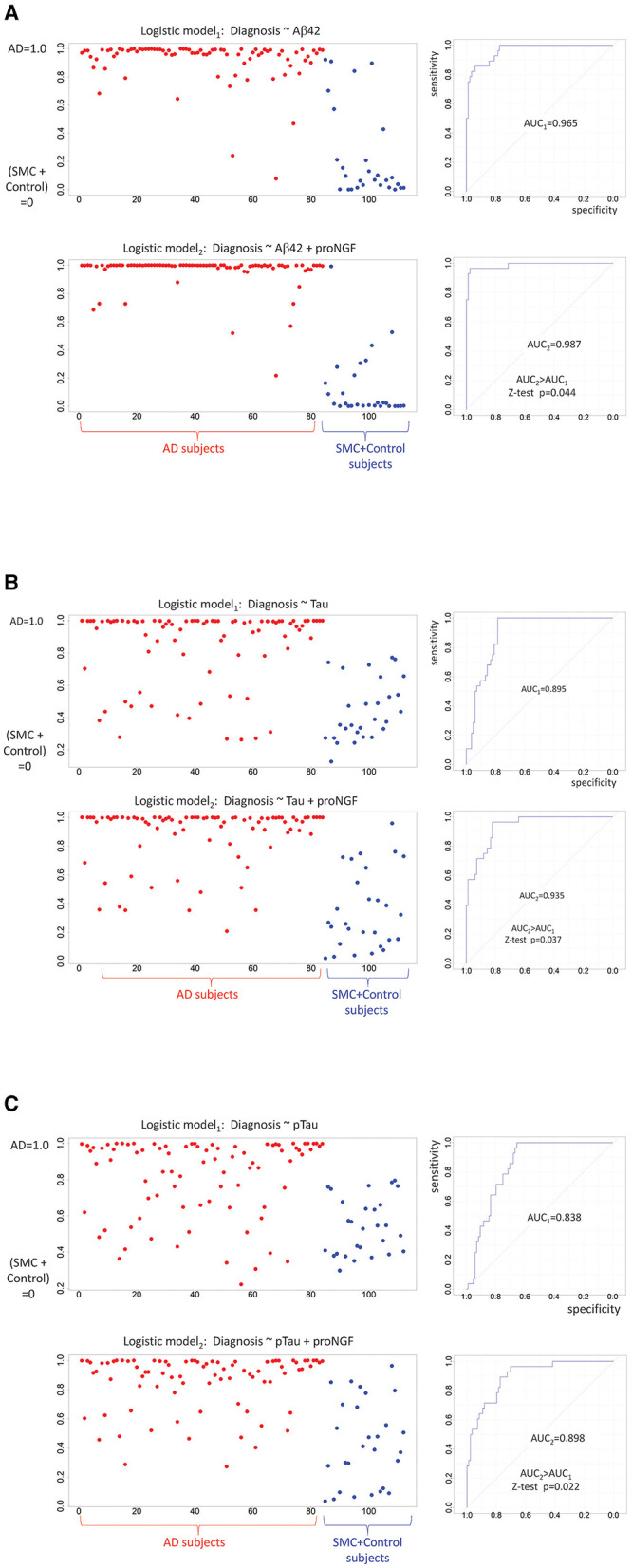
Diagnostic predictions of logistic models: binarized response variable (AD = 1 vs. SMC + control = 0) is predicted by standardized Aβ42 **(A)**, by standardized Tau **(B)**, by standardized pTau **(C)**, and proNGF measures. For each panel, the predicted logistic scores are plotted on the left (AD subjects in red, SMC + control subjects in blue), while the model performances are shown on the right. The two ROC curves are compared by a one-sided *Z*-test (AUC2 > AUC1).

To assess this working hypothesis, we tested the performance of diagnostic models including or not proNGF as a predictor. We compared the logistic regression models using classical biomarkers (e.g., Aβ42, Tau, and pTau) as covariates predicting the cognitive diagnosis (AD = 1 vs. SMC_or_Control = 0), to the same models with the contribution of proNGF as a further covariate: the capacity of Aβ42, Tau, and pTau to predict the cognitive status shows an improvement when proNGF is included as a predictor, as witnessed by the increased AUC (Figure 4).

### Detection of Mature NGF in Human CSF Samples

Even if the concentration of mature NGF in the brain is significantly lower with respect to that of proNGF (Fahnestock et al., 2001; Counts and Mufson, 2005), it would be of interest to determine the levels of mature NGF in CSF. In some (but not all) of the analyzed samples, a peak corresponding to mature NGF, at an apparent MW between 16 and 20 kDa, can be identified (see an example in Supplementary Figure 6). In all the samples in which the mature NGF peak was detected, it was significantly lower with respect to the proNGF peak and, most often, with a low signal-to-noise ratio (see the “Materials and methods” section).

Nevertheless, in some of the analyzed CSF samples, in which the NGF peak was detected (13 AD, 11 SMC, 9 control samples), the areas of the NGF peak exhibited an acceptable signal-to-noise ratio (≥10) and could be analyzed. We tried to carry out an NGF calibration curve by using human recombinant NGF in the experimental conditions optimized for the proNGF immunoassay. However, under these conditions, recombinant NGF was detected at a lower sensitivity than proNGF (see the “Discussion” section).

In Table 3, the median and the average values of the analyzed NGF areas for each group are listed.

**Table 3:** Mean and median of the NGF peaks area for the three diagnostic groups.

	**Diagnosis**	**Area**	**Coefficient of variation (%)**
NGF mean	AD	261,000	16.8
	CTR	379,202	12.0
	SMC	381,741	12.6
NGF median	AD	230,107	15.8
	CTR	371,951	12.2
	SMC	374,729	11.3

Eventually, we analyzed the areas of mature form NGF for reliable measures, finding a pattern very similar to the precursor proNGF (Supplementary Figure 7A), with NGF significantly lower in patients with AD, although the dispersion of data is similar in the three groups (Supplementary Figures 7A,B).

## Discussion

For a long time, proNGF has been considered a promising biomarker for AD (Counts et al., 2016; Cuello et al., 2019; Mufson et al., 2019; Pentz et al., 2020). Importantly, proNGF is a biomarker related to a well-defined neurodegeneration-promoting disease mechanism: the vicious negative loop that causally links alterations in NGF processing and metabolism, leading to an imbalance in the proNGF to NGF ratio, to the activation of neurodegeneration pathways and hallmarks, including Aβ production (Capsoni and Cattaneo, 2006; Bruno et al., 2009; Tiveron et al., 2013; Ioannou and Fahnestock, 2017).

Unfortunately, the validation of proNGF as a biomarker has been hampered by the absence of a reliable and high-throughput immunoassay. It has been demonstrated that NGF and proNGF reciprocally interfere in standard ELISA immunoassays, making the measurements of either protein from *in vivo* samples unreliable and dependent on the unknown proNGF/NGF ratio (Malerba et al., 2016). For these reasons, currently, the only reliable method to measure NGF and proNGF in biological samples that avoids reciprocal interference is immunoblot, a semiquantitative, low sensitivity, and low-throughput method. Moreover, in the literature reports, the measurements of proNGF levels were most often carried out on postmortem brain, the studies were often under-sampled, or were designed primarily for a mere observational research aim, rather than towards the development of a clinical diagnostic tool. To validate proNGF as a diagnostic, prognostic, or stratification biomarker, its levels should be monitored in accessible biological fluids from living patients. The clinically validated biomarkers for AD are all measured in CSF, although significant efforts and resources are being deployed to measure/find biomarkers in less-invasive biological fluids.

So far, only one paper in the literature (Counts et al., 2016) reports a measurement of the proNGF levels by Western blot on postmortem ventricular CSF samples from patients with a diagnosis of mild/moderate AD (*n* = 20), amnestic MCI (*n* = 20), or no cognitive impairment (*n* = 26). It was found that proNGF levels increase during the progression of AD. In that paper, the figure of the Western blot was cut at an MW of about 30 kDa, and only one proNGF band was shown.

We reported here the development and validation of a robust, reliable, automated, and quantitative immunoassay that allows measuring proNGF in the CSF, without NGF interference. So far, this immunoassay is the only one available for measuring proNGF in the CSF of living patients with AD and shows a 40-fold increase in sensitivity with respect to immunoblot performed with the same antibody. By means of this new immunoassay, named Simple ProNGF, we have measured and analyzed the proNGF levels in 84 CSF samples from living patients with AD against 15 SMC subjects and 13 controls.

We found a very significant difference between proNGF levels in AD samples compared with both controls and SMC subjects, while no significant difference was evident between SMC and controls. In the CSF samples from patients with AD, the area of the 34 kDa proNGF peak is about 50% lower compared with that of the other two groups.

More importantly, we found a statistically significant, albeit low, correlation with the currently approved CSF biomarkers for AD. This suggests that proNGF could add new information about the pathology, with respect to the currently validated biomarkers, in addition to being able on its own to discriminate patients with AD from the other diagnostic groups. Moreover, it is noteworthy that when proNGF is included in simple diagnostic binary classifier models with the other biomarkers as covariates, it can improve diagnostic accuracy.

In the MW range between 25 and 50 kDa, three proNGF peak forms were identified. We validated the three peaks as proNGF by means of both Simple Wes and Western blot with two different antibodies, carried out by different procedures (TCA precipitation and immunoprecipitation), and finally confirmed by MS. However, only the band corresponding to the form of proNGF having the same apparent MW of recombinant proNGF from *E. coli*, with no posttranslational modification, was chosen to be further analyzed as a potential biomarker, because it met all the requirements to be reliably measured. Future studies will allow extracting additional information from the analysis and characterization of the other proNGF peaks.

The decreased level of the unmodified “naked form” of proNGF in peripheral CSF from living subjects might be considered unexpected due to the well-established observation of an increased amount of proNGF in the brain homogenates of patients with AD compared with that of the non-demented subjects (Fahnestock et al., 2001; Counts and Mufson, 2005; Al-Shawi et al., 2007; Fahnestock and Shekari, 2019; Pentz et al., 2020).

First of all, our data are the only available on the peripheral CSF of living patients with AD on large sample size. proNGF levels in CSF samples from living patients were never investigated before, under the conditions of our study, due to the absence of a reliable experimental method.

Second, we have demonstrated the identity of three molecular forms of proNGF (also described by other authors, e.g., Lakshmanan et al., 1988; Reinshagen et al., 2000; Pedraza et al., 2005; Kichev et al., 2009; Pentz et al., 2020) by two independent techniques and two different procedures. We have chosen, for further analysis as a candidate biomarker, only the proNGF peak having the same MW of recombinant proNGF. We provide evidence that this form of proNGF is a candidate biomarker for AD and describe the method for measuring it in a reliable and reproducible way. Contrarily, we did not speculate, in this article, about the biological significance of the different proNGF forms, nor did we attempt their molecular characterization. Future work will tell us about the molecular basis for the different proNGF forms we detected and their possible interconversion, and once this will be firmly established, they will provide additional robust biomarkers.

How can we explain the surprising observed decrease of proNGF in the CSF from patients with AD, in face of the well-established increase of proNGF in the parenchyma of Alzheimer's brains? (Pentz et al., 2020).

The observed presence of different proNGF forms, most likely corresponding to different posttranslational modified forms, might explain the decrease of the naked 34 kDa proNGF form. Thus, we could speculate that in AD, there is an increased interconversion of the lower 34 kDa form into higher MW species.

Another clue might come from the case of amyloid-β42 (Aβ42) whose CSF levels often allow to distinguish AD from other dementias and non-demented subjects. Aβ42 is a major component of amyloid plaques and forms oligomeric and fibrillar supramolecular structures (Fagan et al., 2006; Jack et al., 2010). While in the brain of individuals affected by dementia of the Alzheimer's type, Aβ42 is increased, in the CSF, Aβ42 is decreased. This decrease may reflect a “sink effect” by the aggregation process, hindering the transport of soluble Aβ42 between the brain and the CSF (Fagan et al., 2006). To date, we do not know if proNGF exhibits similar behavior, but we have found that proNGF and NGF reciprocally interfere in an immunoassay (Malerba et al., 2016). This issue, on the one hand, has made the development of a reliable immunoassay a complex technical task, but, on the other hand, opens important and interesting biological questions (Malerba et al., 2016). We have hypothesized molecular crosstalk between precursor and mature neurotrophin forms, possibly involving the formation of NGF/proNGF supramolecular structures, similar to the described NGF dimer of dimers (Covaceuszach et al., 2015). Thus, the interplay between the pool of proNGF and NGF in the brain parenchyma with the CSF pool and how this interplay is affected in Alzheimer's brain is interesting and important areas of investigation, possibly involving the formation of supramolecular and intermolecular structures and also involving the pathological posttranslational modifications of proNGF (Pedraza et al., 2005).

Having established this new proNGF assay, numerous open questions still need to be clarified about proNGF as a candidate biomarker for diagnosis or patient stratification: first, whether proNGF is a biomarker that can distinguish between different neurodegenerative diseases or is a specific biomarker for AD, and in this last case, if and how its dosage changes during the progression of the disease. Second, whether proNGF might prove useful as a read-out in clinical trials. Questions that can be answered by this new reliable immunoassay.

Another significant and unexpected result obtained by the analysis of CSF from patients was the detection of mature NGF peaks. Since it is well-known from the literature that NGF is significantly less concentrated compared with its precursor in the brain (Fahnestock and Shekari, 2019), the NGF detection was not foregone, also because our immunoassay was optimized for proNGF recognition. Despite the lower number of samples in which the NGF peak could be detected unambiguously (but not lower compared with the usual sample size commonly reported in the literature), we demonstrated that also NGF levels (similarly to proNGF levels) were significantly lower in AD samples compared with both controls and SMC subjects, while no significant difference was evident between SMC and controls.

Also, the ability to measure NGF is very relevant, because the proNGF/NGF ratio might be even more informative than the absolute levels of proNGF, being directly linked to neurodegeneration mechanisms (Capsoni et al., 2011; Schliebs and Arendt, 2011; Tiveron et al., 2013; Arisi et al., 2014; Mufson et al., 2019). It is, therefore, of interest to discuss the case of the samples where NGF could not be reliably estimated, though being clearly present since a small peak could be detected in electropherograms. The proNGF/NGF ratios for the *n* = 33 samples reliably measured fall within the range 2.14–6.72, with a maximum CV = 46%. Furthermore, we estimated the smallest value for NGF peak areas that we could reliably measure and refer to this value as NGF_min_. We can thus estimate a lower limit for the proNGF/NGF ratios of the *n* = 79 samples where the NGF peaks were below the threshold, being too small to be reliably quantified as proNGF/ NGF_min_. With these assumptions, the ratios for these *n* = 79 samples fall within the range of 1.91 ± 0.87–14.47 ± 6.62. We can therefore argue that the “true” proNGF/NGF ratio is likely to reach higher values (>14) for these subjects, with an overabundance of proNGF. It will be of interest to determine whether these patients might represent a clinically identifiable subgroup.

Coherently with the significantly larger NGF levels in controls (CTR) or SMC group compared with AD subjects (Supplementary Figure 7A), comparing the number of samples where NGF could be reliably detected in AD samples (13/84) and CTR+SMC samples (20/28), we observed a significantly higher proportion in CTR + SMC subjects (Fisher's exact test *p*-value = 8.21^*^10–8, odds ratio = 13.6).

In the future, we aim to further optimize the assay to obtain a multiplex measure of both NGF and proNGF, with the goal of measuring the proNGF/NGF ratio in the Alzheimer's continuum and for the differential diagnosis in different neurodegenerative diseases.

This immunoassay could also be applied to explore in deeper detail the role of NGF precursor in the pathogenesis of neurodegenerative diseases and in physiological conditions.

### Limitations of the Study

The main limitation of the study is that we did not analyze the higher MW proNGF peaks. This issue is justified by the fact that to obtain a detectable signal, CSF samples must be concentrated, and only the peak corresponding to the MW of 34 kDa gave consistent results between the TCA concentrated and the neat samples, as described above. Instead, the data for the higher MW peaks (39 and 45–50 kDa) gave inconsistent results, with respect to the dilution ratio after concentration. Despite this limitation, the main aim of this study was reached, namely, the identification and validation of a candidate biomarker for AD. Indeed, the 34 kDa proNGF form, which exhibits the same MW of the recombinant proNGF, had all the features to be a candidate biomarker for AD. Contrarily, the lack of reliable measures for higher MW proNGF peaks did not allow investigation of the biological value of these modified forms and their possible relation to the disease process. One way to overcome this limitation could be to avoid the concentration of the biological samples by improving the sensitivity of the method. A more sensitive method could also help to obtain a higher signal for the NGF peaks necessary for robust measurement of the proNGF/NGF ratio, which is considered another potential biomarker of neurodegeneration (Capsoni et al., 2011; Schliebs and Arendt, 2011; Tiveron et al., 2013; Arisi et al., 2014; Mufson et al., 2019). The next step will be indeed to try to improve the sensitivity of the Simple ProNGF assay.

Another limitation of the study is the small number of samples for the control and SMC groups with respect to the AD group. Having CSF from living non-demented patients is not straightforward, due to the fact that CSF withdrawal is invasive and neither patients nor ethical committee authorize this procedure if not strictly necessary for the subject and required by clinical protocols; therefore, non-demented subjects are less likely to undergo a lumbar puncture. The sample size difference between the groups makes the one-way ANOVA design analysis a bit unbalanced, but anyway, the Kruskal–Wallis test achieves a very large statistical Power for proNGF (0.97) and is good for NGF (0.84), while the significant *post-hoc* tests achieve Power 0.94 (AD-CTR) and 0.99 (AD-SMC) for proNGF and 0.41 (AD-CTR) and 0.56 (AD-SMC) for NGF. Increasing the sample size for control and SMC groups will certainly improve the NGF analysis. The Power was computed using G^*^Power.

Another limitation is the difference in age (about 10 years) between the group of SMCs and the other groups. Memory complaints are present in adults of all ages, but, in general, the SMC subjects are younger than mild cognitive impairment and AD dementia subjects, because younger subjects are more likely to report a cognitive complaint as pathological or worrisome, especially if related to memory. The SMC is a relatively new diagnostic category that has been recently identified (Jessen et al., 2014), with a wide variety of instruments and domains (memory domains are generally the most investigated followed by executive function and attention) (Rabin et al., 2015). However, in younger subjects, the cognitive or memory complaints are less likely to be related to objective cognitive impairment and neurodegeneration, and this makes less likely an association with a specific biomarker of neurodegeneration. Given the challenge due to heterogeneity, this is, however, a group of subjects that has to be investigated in studies on neurodegeneration because it will help to identify an earlier stage of AD.

## Data Availability Statement

The datasets presented in this study can be found in online repositories. The dataset LC-MS/MS analysis of NGF proteins purified from cerebrospinal fluid for this study can be found in the Pride. (http://www.ebi.ac.uk/pride). Project accession: PXD025883; Project doi: 10.6019/PXD025883.

## Ethics Statement

The studies involving human participants were reviewed and approved by Center for Neurodegenerative Diseases and the Aging Brain of the University of Study of Bari Aldo Moro at Pia Fondazione Card. Panico Hospital (Tricase). The patients/participants provided their written informed consent to participate in this study.

## Author Contributions

FM, RF, BBE, CZ, MD'A, SC, and MC performed the experiments. IA carried out the statistical analysis. CZ, MD'A, and GL recruited the patients. FM and AC designed the study with the support of GL. FM, IA, and AC wrote the article. All authors contributed to the article and approved the submitted version.

## Funding

This work was supported by the European Commission (MADIA no. 732678), by Regione Lazio (Public Notice LIFE 2020, MODIAG Project), by Fondo Ordinario Enti (FOE D.M 865/2019) in the framework of a collaboration agreement between the Italian National Research Council and EBRI (2019–2021), and by Puglia Region (CUP B84I18000540002 TecnoMed Puglia Establishment of the TECNOPOLO for Precision Medicine). The authors acknowledge Banca d'Italia and Chiesi Farmaceutici for the donations supporting the project. The authors also thank the financial support of Istituto Neurologico Carlo Besta (Programma Strategico di Ricerca Finalizzata, 2007).

## Conflict of Interest

The authors declare that the research was conducted in the absence of any commercial or financial relationships that could be construed as a potential conflict of interest.

## Publisher's Note

All claims expressed in this article are solely those of the authors and do not necessarily represent those of their affiliated organizations, or those of the publisher, the editors and the reviewers. Any product that may be evaluated in this article, or claim that may be made by its manufacturer, is not guaranteed or endorsed by the publisher.
