# Supplementary material for: proNGF Measurement in Cerebrospinal Fluid Samples of a Large Cohort of Living Patients With Alzheimer's Disease by a New Automated Immunoassay
Source: Front Aging Neurosci. 2021 Oct 27;13:741414. doi: 10.3389/fnagi.2021.741414 (PMC8579109; doi:10.3389/fnagi.2021.741414)
Supplement: Supplementary file 1 [file Data_Sheet_1.docx]

Supplementary Material

# Material and Methods

**Patients enrollment: Medical examination**

Demographic data included age, sex, and years of education were collected. A structured interview exploring familiar, personal and medical history, social status and overall physical exam was performed (Maggi et al., 1994; Scafato et al., 2005). For each patient, a neurological examination was carried out including the Clinical Dementia Rating Scale (CDR), a tool designed to grade subjects from normal function through various stages of dementia (Hughes et al., 1982) and . the Mini Mental State Examination (Folstein et al., 1975) in order to evaluate the impairment of global cognition and of specific cognitive domains.

Patients diagnosed with SMC presented subjective memory concern and were “self-referrals”. Criteria for diagnosis were: 1) self-experienced persistent decline in memory and cognitive capacity in comparison with a previously normal status and unrelated to an acute event; 2) normal age-, gender-, and education-adjusted performance on standardized cognitive tests, which are used to classify mild cognitive impairment (MCI) or prodromal AD (Jessen et al., 2014). CDR = 0 and MMSE score between 24 and 30 (inclusive) were considered for this group.

Patients with AD were diagnosed using standard diagnostic criteria for dementia (Diagnostic and Statistical Manual of Mental Disorders, 5th ed. (DSM-5) (Association, 2013), and National Institute on Aging-Alzheimer’s Association (NIA-AA) criteria (McKhann et al., 2011). Patients included in this group had CDR score equal 1 and the MMSE score <24.

The subjects of the control group were cognitively normal without any subjective complaint, based on an absence of significant impairment in cognitive functions or activities of daily living. They were selected according to ADNI-3 criteria (Weiner et al., 2017). no signs of depression, mild cognitive impairment, or dementia; normal memory function documented by scoring above education adjusted cutoffs on the Logical Memory II subscale (Delayed Paragraph Recall, Paragraph A only) from the Wechsler Memory Scale-Revised; MMSE score between 24 and 30. A summary of the inclusion criteria is shown in the Table 1.

**Table 1:** Inclusion criteria of the subjects enrolled in the study

| **AD** | **SMC** | **CONTROLS** |
| --- | --- | --- |
| DSM-5 / NIA-AA criteria for AD dementia | Subjects "self-referrals" of subjective memory concern, but cognitive complaints and tests did not meet the criteria for dementia or other neurological or psychiatric disorder | Cognitively normal without any subjective complaint, based on an absence of significant impairment in cognitive functions or activities of daily living |
| MMSE score <24 | MMSE score 24 - 30 (inclusive) | MMSE score 24 - 30 (inclusive) |
| CDR= 1 | CDR= 0 | CDR= 0 |

**Cerebrospinal fluid biomarkers analysis**

All patients underwent the lumbar puncture according to standard procedures. The cerebrospinal fluid (CSF) sample was centrifuged at room temperature for ten minutes at 2000g (rcf), aliquoted and stored at −80°C until analysis, according international biomarkers recommendations (Vanderstichele et al., 2012).

The CSF Aβ42, Tau and p-Tau181 levels were measured by chemiluminescent immunoassay CLEIA (Lumipulse G ß- amyloid 1–42, Lumipulse G Total Tau, Lumipulse G pTau181, Fujirebio Europe N.V., Gent, Belgium) on fully automatic platform (Lumipulse G600II, Fujirebio Europe N.V., Gent, Belgium). All the assays were performed according to manufacturer's protocols.

For the interpretation of the cerebrospinal biomarker results, the following cut-off values were considered: Aβ42 >683 pg/mL, Tau <342 pg/mL, p-Tau181 <57 pg/mL. Consistent with the diagnostic criteria for Alzheimer’s disease (Dubois et al., 2014), a cerebrospinal fluid biomarker profile was considered to be suggestive for Alzheimer’s disease if the CSF Aβ1–42 value was below the cutoff, in combination with T-tau and/or P-tau181 values above the threshold.

**Validation of the immunoassay**

The assay was validated firstly with artificial cerebrospinal fluid spiked with human recombinant proNGF, and then with eight cerebrospinal fluid samples from patients.

In order to check the cross-reactivity of the assay, the same cerebrospinal fluid sample was run with and without the primary or the secondary antibody. Moreover, Simple Wes Fluorescent Master mix were run in absence of cerebrospinal fluid sample.

In order to evaluate the effect of sample processing, three cerebrospinal fluid samples with a large amount of proNGF were selected and run in Simple Wes side by side, both neat and concentrated, as described in the method section (see manuscript). For each measurement, the area under the curve of the concentrated sample was divided by the area of the same sample, run neat. The measured ratios were compared to the expected ratio value (13) by the one sample 2-sided t-test, by the mean of the web tool available at the link: <https://www.graphpad.com/quickcalcs/oneSampleT2/>.

The robustness of the assay was evaluated by testing the same sample in different assays and in different days. Nine independent measurements for two cerebrospinal fluid samples were carried out. A CV≤20% was considered acceptable.

In order to establish the sensitivity of the assay, the limits of detection for recombinant human proNGF in Simple Wes and in Western Blot were compared. Western Blot was carried out by loading decreasing amount of human recombinant proNGF (from 800 to 6.2 ng per lane). The membrane was challenged by the same antibody used in Simple Wes: MyBiosource anti NGF MBS125020, at the work concentration 1:100, incubated overnight at 4°C, while the secondary antibody was goat anti-rabbit HRP conjugated (Jackson, West Grove, PA, USA), incubated at room temperature for 1 hours. The immunoblot was developed with the ECL Advance substrate (Cytiva, RPN418) by the mean of the iBright™ CL1500 Imaging System (see figure 2).

**Identification of proNGF peaks**

An immuno-depletion experiment with an anti NGF antibody was carried out. A CSF sample from the same patient was divided into two aliquots of 130 µL. One aliquot was processed normally, the other one was immunoprecipitated by αD11 (Cattaneo et al., 1988), the monoclonal antibody able to recognize a specific epitope of the mature NGF, and to immunoprecipitate both NGF and proNGF (Tiveron et al., 2013). 20 µL of Protein G resin was conjugated with 1 µg of aD11 for 4 hours at 4°C on a rotating wheel. The resin was washed and then incubated with 130 µL of CSF sample overnight on a rotating wheel. The resin was centrifuged at 3000 rpm for 3 minutes and the supernatant was recovered and processed as previously described. The immune-deprived and the normal processed samples were run side by side, using the same antibodies and run parameters.

**Mass Spectrometry analysis**

**Sample Preparation**

A fixed volume from 8 cerebrospinal fluid samples of patients was pooled. Mab αD11 (Cattaneo et al., 1988) was conjugated and crosslinked to Protein G Sepharose. In details, in a unique vial, an amount of Protein G Sepharose enough to obtain 20 µL for each sample was washed by sodium tetraborate 0.1M pH 9. 2 µg of Mab αD11 for sample were added to the resin and incubated overnight at 4°C on a rotating wheel. The resin was washed, resuspended in sodium tetraborate 0.1M pH 9 containing 20 mM of DMP (dimethyl pimelimidate, ThermoFisher) and then incubated on a rotating wheel at room temperature. The resin was washed, resuspended in Tris-HCl 50 mM pH 7.5 and incubated 2 hours at room temperature, in order to quench the reaction. The resin was then washed in PBS.

260 µL of the CSF pool, 10 µg of recombinant human NGF, 10 µg of recombinant human proNGF, were immunoprecipitated overnight. The resins were washed, resuspended in Laemli buffer, boiled and then loaded on a precast gel (Bio-Rad criterion, 4–12% BIS-Tris, 12+2). As control, also an aliquot from the supernatant of the immunoprecipitation samples, containing the CSF compounds that did not bind αD11, was loaded on the gel. EZWay Protein-Quick Blue staining solution (K14050) was used to stain the gel. The bands detected in the lane corresponding to the CSF pool were analyzed in Mass Spectrometry. The experiment was performed three times with three independent pools.

A control western blot was also carried out. 130 µL of the cerebrospinal fluid pool, 3 µg of recombinant human NGF mixed to 3 µg of recombinant human proNGF, were immunoprecipitated overnight, by using the same Mab αD11 crosslinked to resin, used for the SDS-PAGE. The resin was processed as previously described, and the samples were divided into two aliquots and loaded twice on a precast gel (Bio-Rad criterion, 4–12% BIS-Tris, 12+2) and then transferred to a nitrocellulose membrane in western blotting. The membrane was cut and half was challenged with ANT 005 anti-proNGF (working concentration 1:200; Alomone, Jerusalem, Israel) and the other half with MBS125020 anti NGF (working concentration 1:100; myBiosource). The secondary antibody was goat anti-rabbit HRP conjugated (Jackson Immunoresearch) for both the western blots. The immunoblots were developed with the ECL Advance substrate (Cytiva, RPN418) by the mean of the iBright™ CL1500 Imaging System (see figure 4).

**Mass Spectrometry and bands analysis**

The Coomassie stained band were cut, destained with a solution containing 50mM bicarbonate ammonium (AMBIC) and acetonitrile (1:1), incubated with 10 mM dithiothreitol at 56°C for 45min, washed, incubated with 55 mM iodoacetamide at room temperature for 30 min, dried with acetonitrile and then in a Speed Vac and, finally, treated with 12.5ng/µl trypsin (Promega Corporation, WI, United States) in 25 mM AMBIC overnight at 37°C. The peptide mixture obtained was analyzed by liquid chromatography-mass spectrometry (LC-MS) analysis using with an Ultimate 3000 HPLC (Dionex, Thermo Fisher Scientific) on-line with an Orbitrap Fusion Tribrid (Thermo Fisher Scientific, CA, United States) mass spectrometer. Peptides were desalted on a trap column (Acclaim PepMap 100 C18, Thermo Fisher Scientific) and then separated onto a 45-cm-long silica capillary (Silica Tips FS 360-75-8, New Objective, MA, United States), packed in-house with a C18, 1.9μm size particle (Michrom BioResources, CA, United States). A 60 min gradient was applied using buffer A (95% water, 5% acetonitrile, and 0.1% formic acid) and B (95% acetonitrile, 5% water, and 0.1% formic acid): B buffer was increased from 5% to 30% in 35 min, then to 80% in 5 minutes, and finally the column was washed and equilibrated again for the following run. MS spectra were acquired in the orbitrap at 120k while MS/MS spectra were acquired in the linear ion trap.

Spectra were analyzed using the software Proteome Discoverer 2.4 (Thermo) using human and rat databases downloaded from Swiss Prot (https://www.uniprot.org/).

Parameters used for the searches were: 10 ppm and 0.6 Da tolerance for precursor and fragment ions, respectively, specific tryptic cleavage with two missed cleavages allowed, fixed cysteine carbamidomethylation, and variable methionine oxidation. Percolator node was applied to obtain protein identification at 0.01 FDR. MS data are available via ProteomeXchange with identifier PXD025883(Perez-Riverol et al., 2019).

# Supplementary Figures

**Figure 1.** Electropherograms obtained from the cross-reactivity experiment.


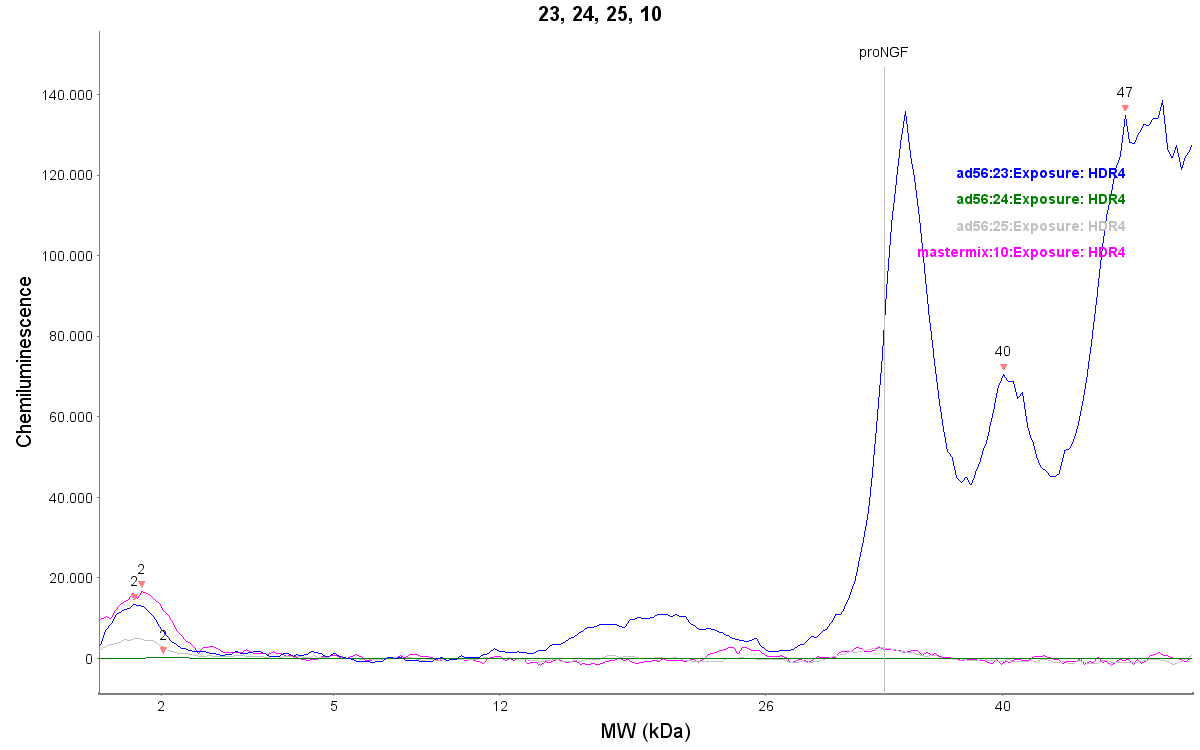


**Figure 1.** Electropherograms obtained from the cross-reactivity experiment. The same CSF sample from a patient with AD was run normally (blue electropherogram), without the primary antibody MBS125020 (grey electropherogram), or without the secondary biotinylated antibody (green electropherogram). Moreover, Simple Wes Fluorescent Master mix were run in absence of CSF sample (pink electropherogram).

**Figure 2.** Limit of detection for recombinant human proNGF in Western Blot (MyBiosource anti NGF MBS125020)


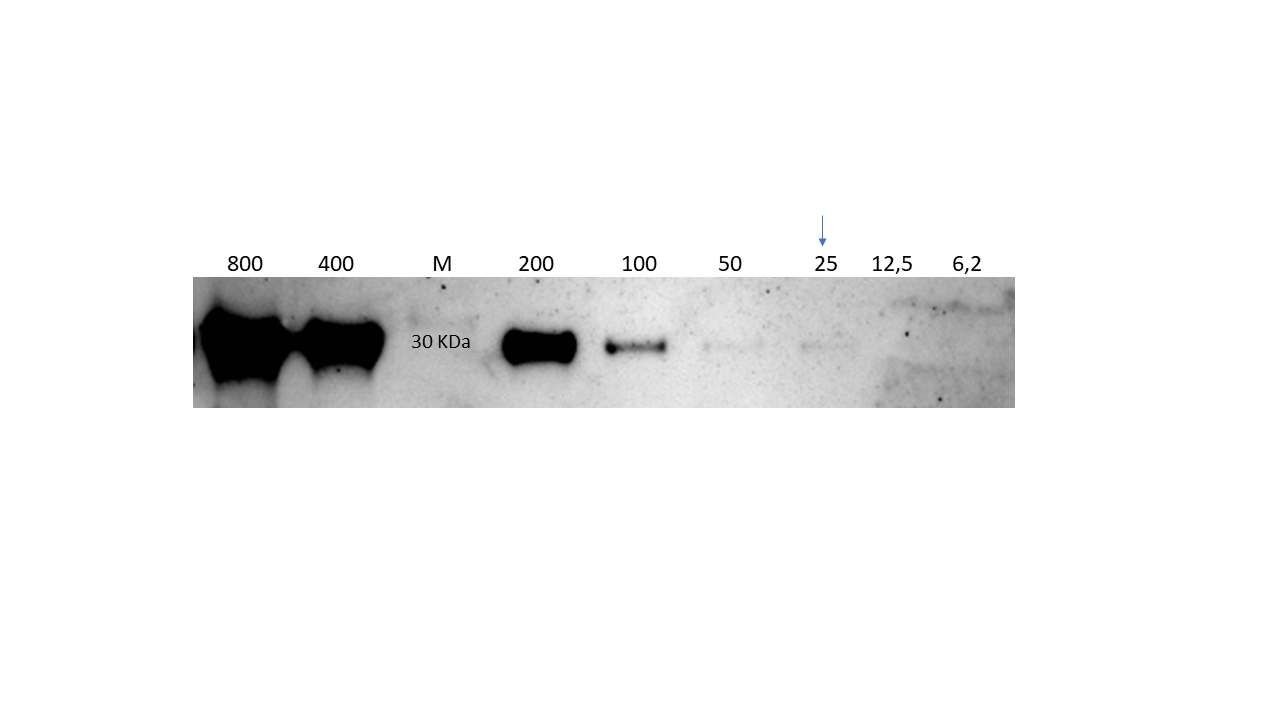


**Figure 2.**  The Western Blot was carried out by loading 800 - 400 – 200 -100 – 50 – 25 – 12.5 – 6.2 ng per lane of human recombinant proNGF. The membrane was challenged by MyBiosource anti NGF MBS12502. In the third position, the “Novex Sharp Pre-stained Protein Standard” (Invitrogen) was used as molecular weight marker (M).

**Figure 3.** SDS-PAGE for Mass Spectrometry (MS) experiment.


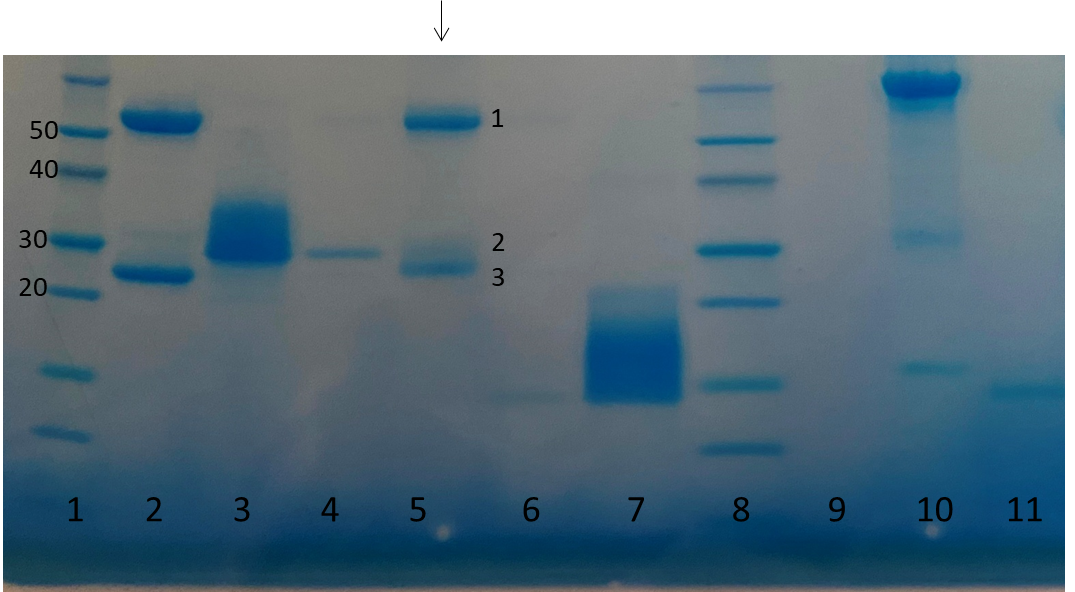


**Figure 3.** A representative SDS-PAGE for MS experiment. Several immunoprecipitation experiments were carried out by αD11 anti-NGF monoclonal antibody as described in the supplementary methods sections, and both the immunoprecipitated and the supernatants were run on SDS- PAGE. Loading order. Lanes: ***1, 8*** Novex Sharp Pre-stained Protein MW Marker; ***2*** anti NGF monoclonal antibody αD11 10 µg; ***3*** recombinant human proNGF 10 µg; ***4*** αD11 immunoprecipitation of 10 µg of recombinant human proNGF; ***5*** αD11 immunoprecipitation of a pool of CSF sample from eight AD patients; ***6*** αD11 immunoprecipitation of 10 µg of recombinant human NGF; ***7*** recombinant human NGF 10 µg; ***9*** supernatant (unbound) of the αD11 immunoprecipitation of 10 µg of recombinant human proNGF (lane 4); ***10*** supernatant (unbound) of the αD11 immunoprecipitation of a pool of CSF sample from eight AD patients (lane 5); ***11*** supernatant (unbound) of the αD11 immunoprecipitation of 10 µg of recombinant human NGF (lane 6).

**Figure 4.** Western Blot to confirm SDS-PAGE for Mass Spectrometry (MS) experiment


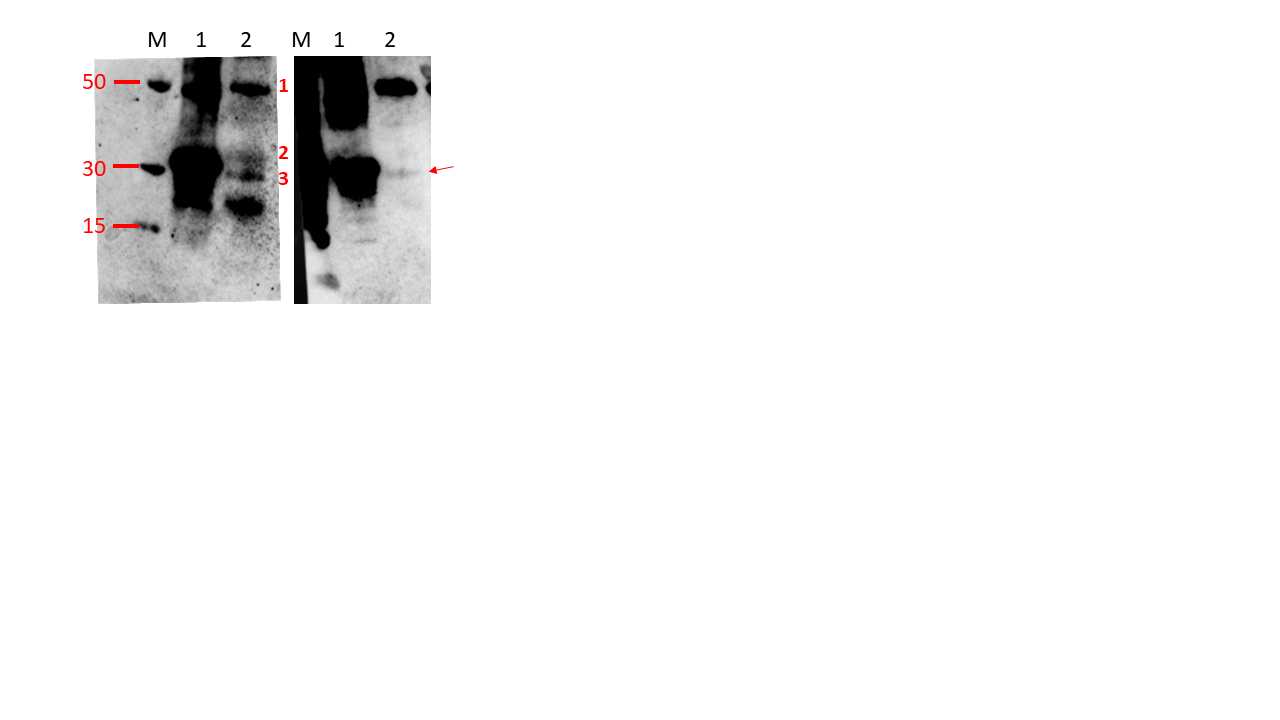


**B**

**A**

**Figure 4.** A representative Western Blot to confirm proNGF bands in the SDS-PAGE, used for MS experiment. 130 µL of the cerebrospinal fluid pool and 3 µg of recombinant human NGF mixed to 3 µg of recombinant human proNGF, were immunoprecipitated by using the same reagents and protocol, used for the SDS-PAGE. The samples were divided into two aliquots and loaded twice on a precast gel (Bio-Rad criterion, 4–12% BIS-Tris). Loading order. Lanes: ***M*** Novex Sharp Pre-stained Protein MW Marker; ***1*** αD11 immunoprecipitation of 3 µg of recombinant human proNGF + 3 µg of recombinant human NGF; ***2*** αD11 immunoprecipitation of a pool of CSF sample from eight AD patients. The membrane was cut and half was challenged with (**A**) ANT 005 anti-proNGF (working concentration 1:200; Alomone, Jerusalem, Israel) and the other half with (**B**) MBS125020 anti NGF (working concentration 1:100; myBiosource). As evident from the figure, ANT005 is more efficient than MBS125020 in detecting the proNGF band pattern, while MBS125020 is able to reveal two out of three of the proNGF bands (30 and 50 KDa), despite the long exposition time. This is probably due to its low sensitivity in Western Blot.

**Figure 5.** ProNGF identification by LC-MS/MS

**A**

**
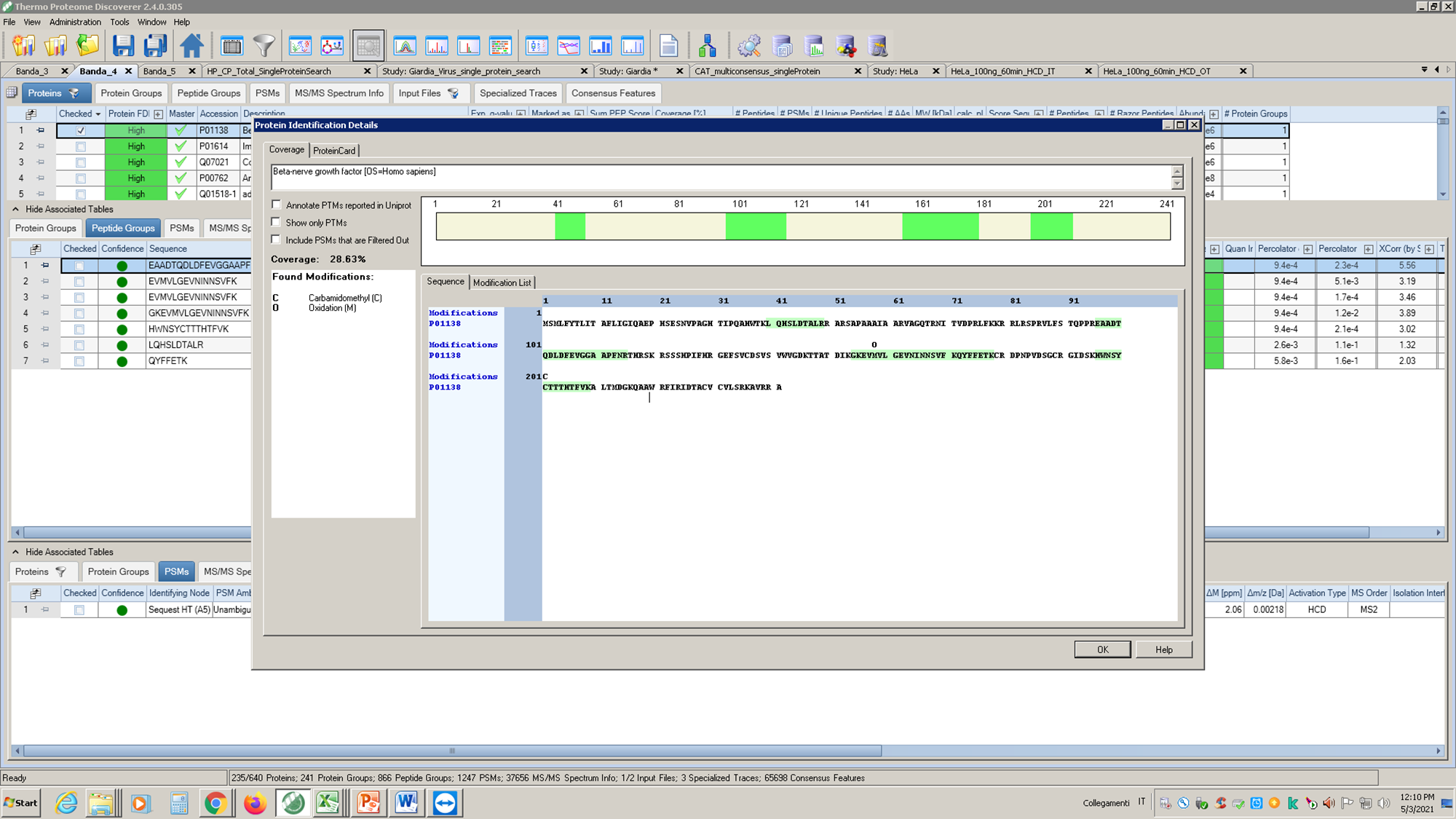
**

**B**

**
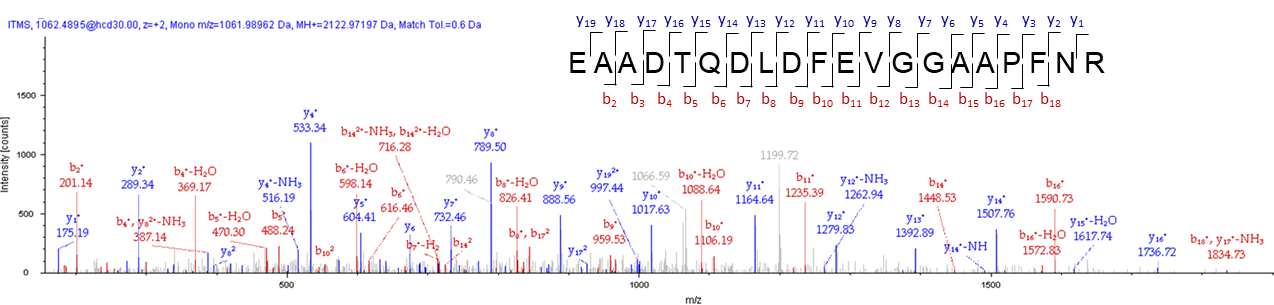
**

**C**

**
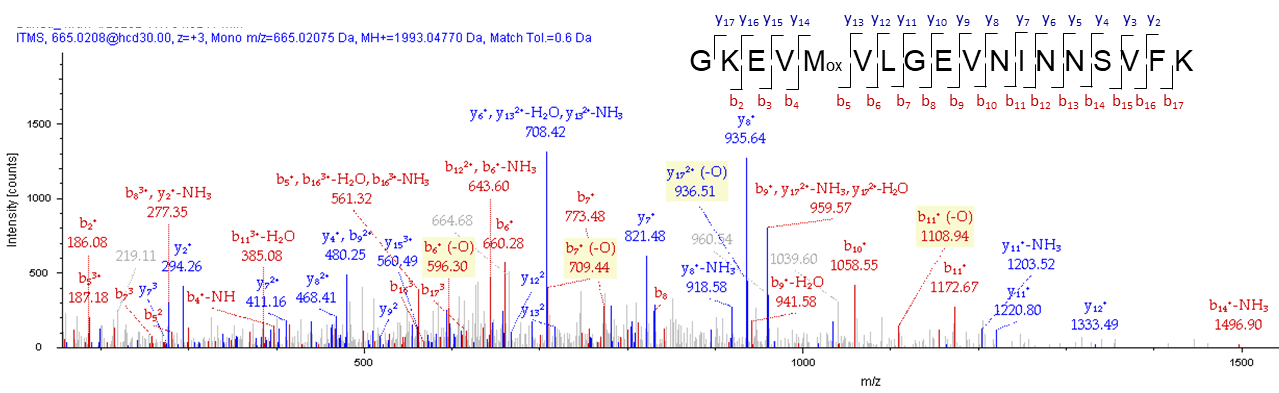
**

**D**

**
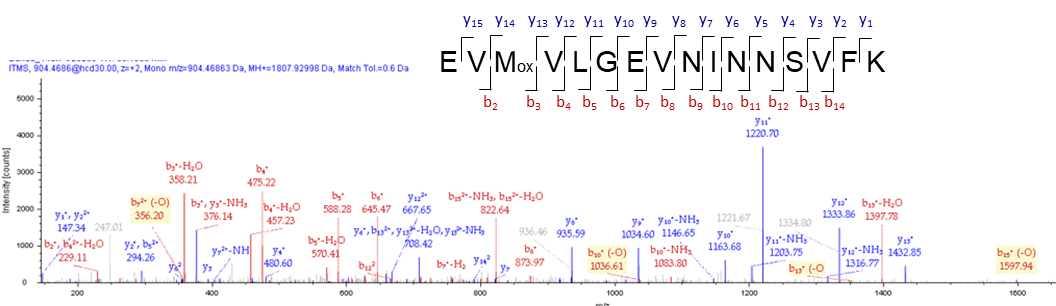
**

**E**

**
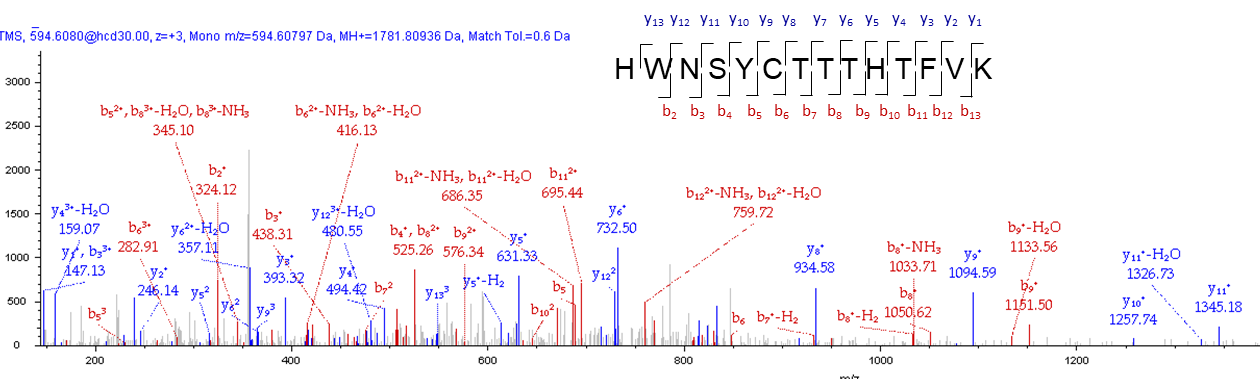
**

**F**

**
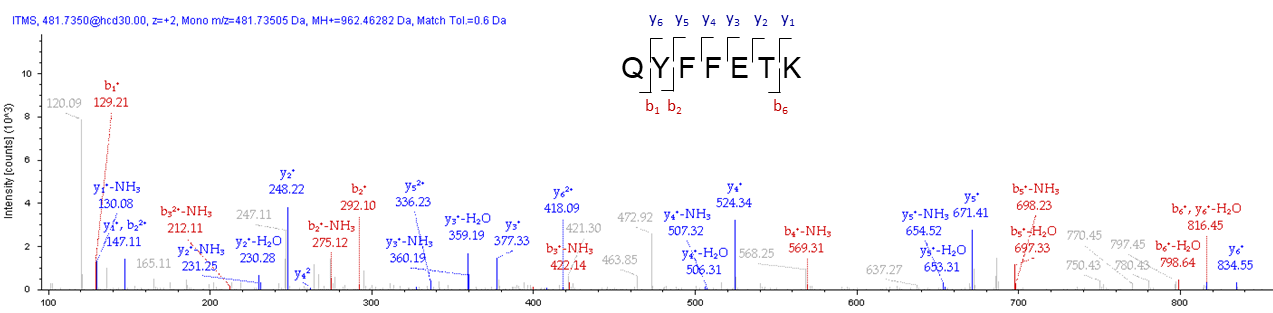
**

**G**

**
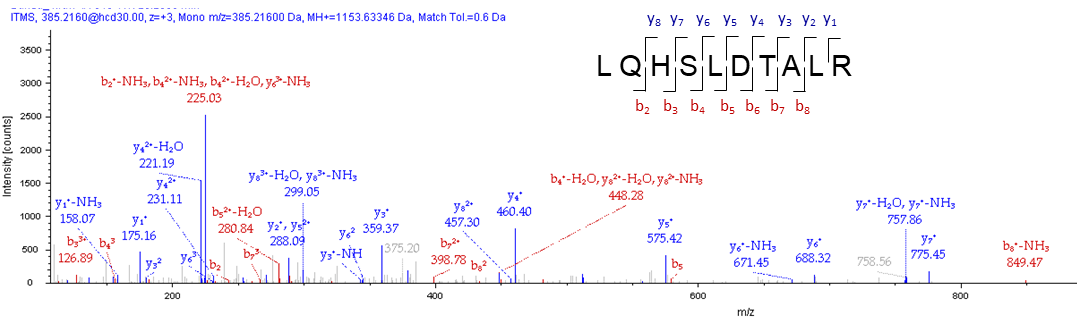
**

**Figure 5.** ProNGF identification by LC-MS/MS **A**. ProNGF protein sequence coverage: Green highlighted peptides are experimentally matched by LC-MS/MS analysis**. B-G**. MS/MS spectra of the matched peptides: EAADTQDLFEVGGAAPFNR with double charged precursor ion at m/z 1061.99 (**B)**; GKEVMVLGEVNINNSVFK with triple charged precursor ion at m/z 665.02 (**C**); EVMVLGEVNINNSVFK with double charged precursor ion at m/z 904.47 (**D**); HWNSYCTTTHTFVK with triple charged precursor ion at m/z 594.61 (e); QYFFETK with double charged precursor ion at m/z 481.74 (**F**); LQHSLDTALR with triple charged precursor ion at m/z 385.22 (**G**). b- and y-series ions are shown in each MS/MS spectrum in red and blue, respectively. The sequence of the peptide matching the fragments detected in each MS/MS spectrum is reported in the inner panel.

**Figure 6.** A representative electropherogram of an AD CSF sample in which the peak of mature NGF (at 16 KDa) is well represented.


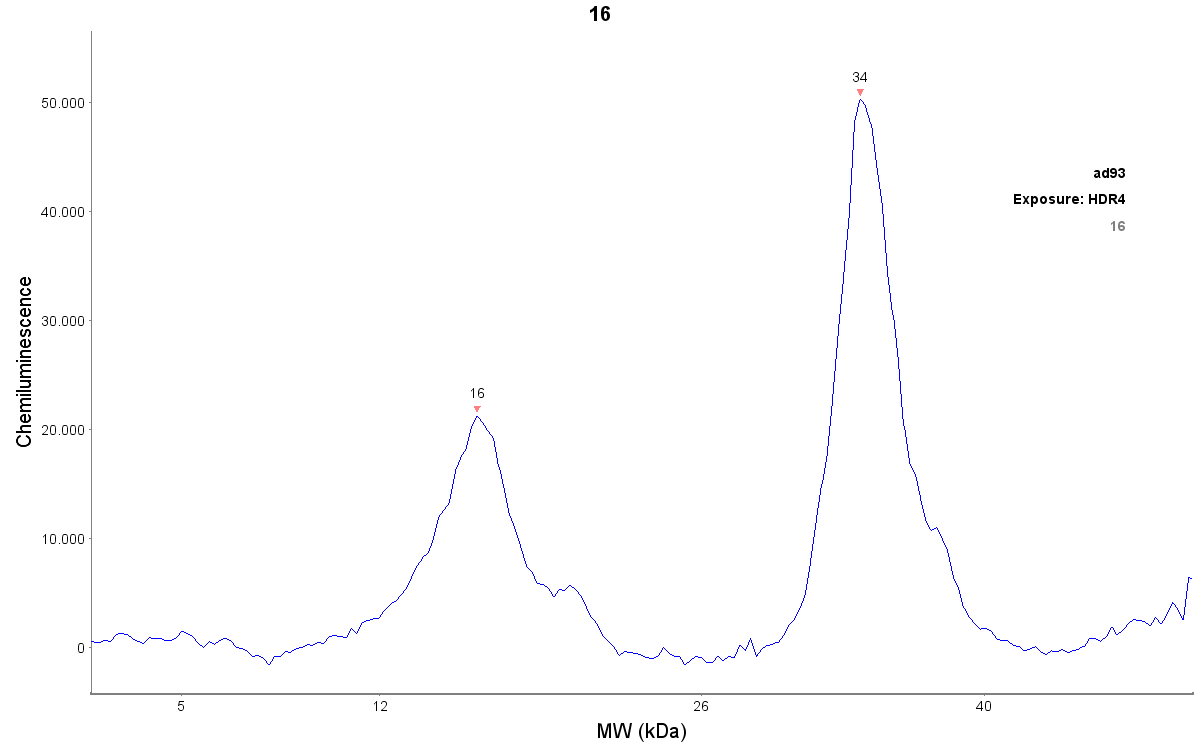


**Figure 7.** Statistical analysis of NGF peak areas in the 3 diagnostic group

**A**

**
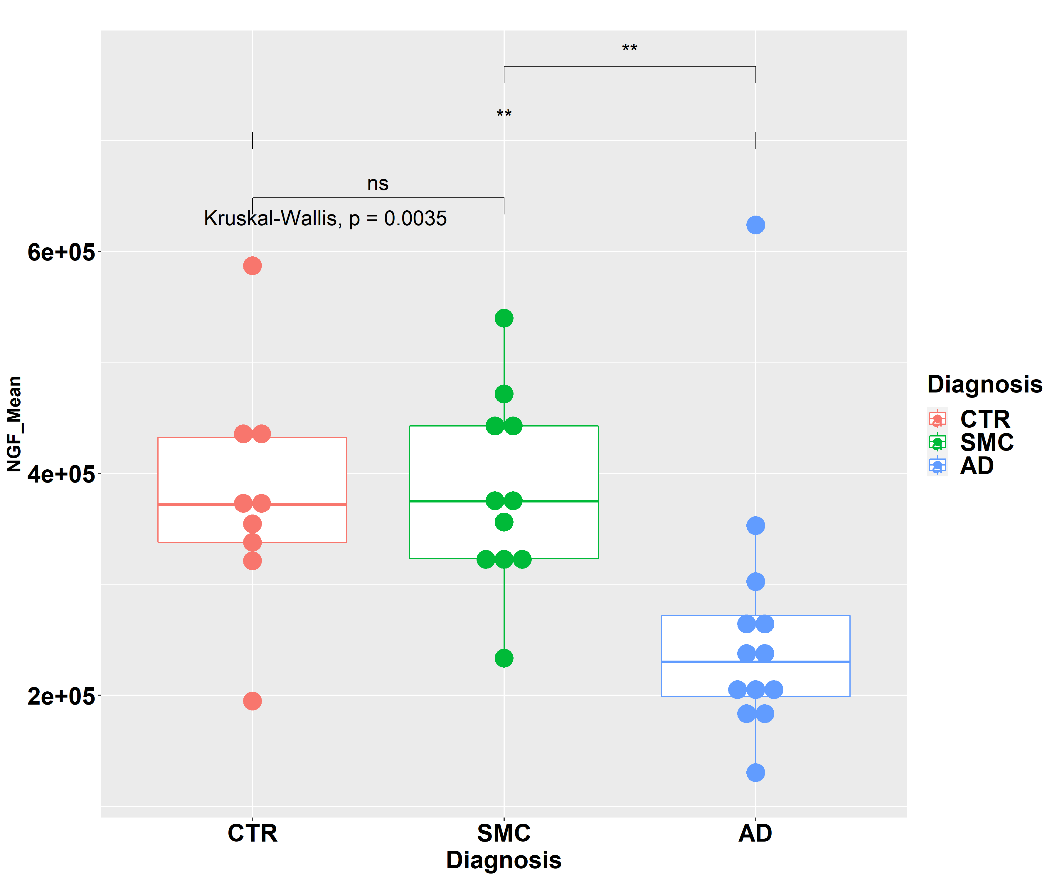
**

**B**

**
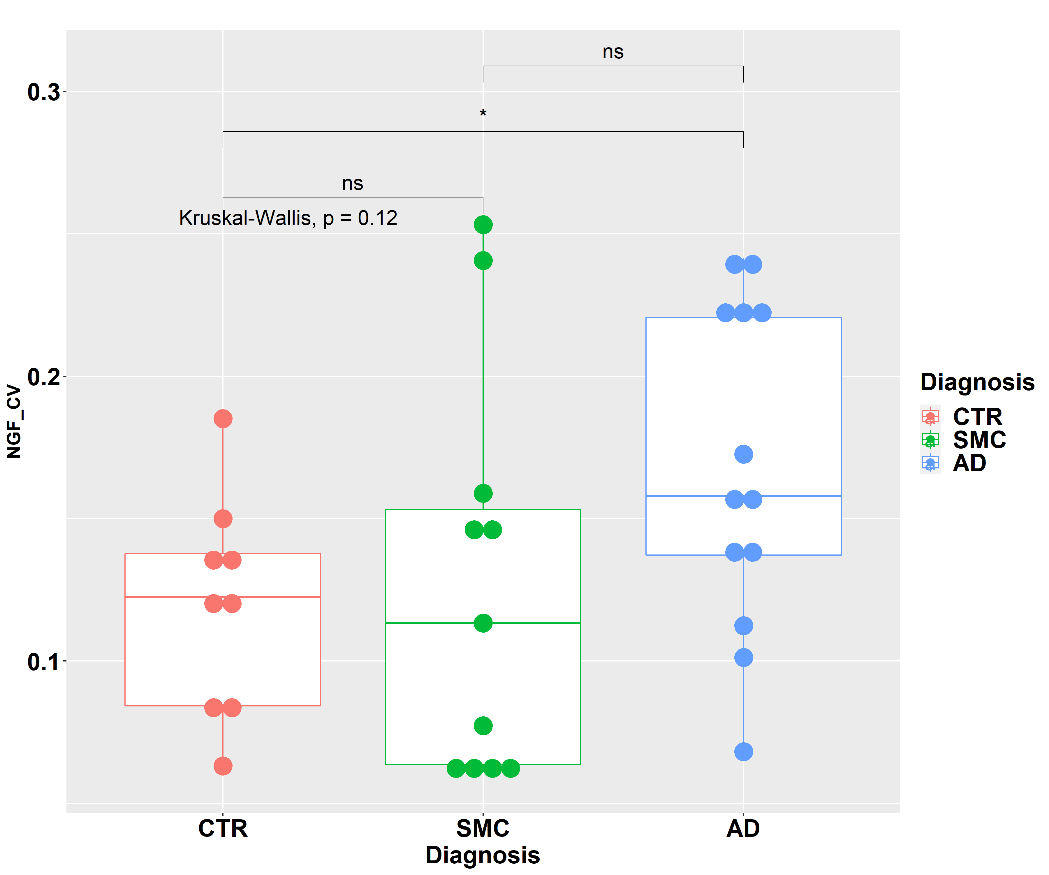
**

**Figure 7. A.** Measure of NGF (peak area) in the three diagnostic groups. Kruskal-Wallis test is followed by pairwise Mann-Withney test with P-value correction (horizontal bars). The difference in data dispersion between groups was analyzed by the Variance F-test. **B.** Coefficient of variation (SD/mean) for NGF measures in the three diagnostic groups. Kruskal-Wallis test is followed by pairwise Mann-Withney test with P-value correction (horizontal bars).

**References**

Association, A.P., 2013. Diagnostic and statistical manual of mental disorders (DSM-5®). American Psychiatric Pub.

Cattaneo, A., Rapposelli, B., Calissano, P., 1988. Three distinct types of monoclonal antibodies after long‐term immunization of rats with mouse nerve growth factor. J. Neurochem. 50, 1003–1010.

Dubois, B., Feldman, H.H., Jacova, C., Hampel, H., Molinuevo, J.L., Blennow, K., DeKosky, S.T., Gauthier, S., Selkoe, D., Bateman, R., 2014. Advancing research diagnostic criteria for Alzheimer’s disease: the IWG-2 criteria. Lancet Neurol. 13, 614–629.

Folstein, M.F., Folstein, S.E., McHugh, P.R., 1975. “Mini-mental state”: a practical method for grading the cognitive state of patients for the clinician. J. Psychiatr. Res. 12, 189–198.

Hughes, C.P., Berg, L., Danziger, W., Coben, L.A., Martin, R.L., 1982. A new clinical scale for the staging of dementia. Br. J. Psychiatry 140, 566–572.

Jessen, F., Amariglio, R.E., Van Boxtel, M., Breteler, M., Ceccaldi, M., Chételat, G., Dubois, B., Dufouil, C., Ellis, K.A., Van Der Flier, W.M., 2014. A conceptual framework for research on subjective cognitive decline in preclinical Alzheimer’s disease. Alzheimers Dement. 10, 844–852.

Maggi, S., Zucchetto, M., Grigoletto, F., Baldereschi, M., Candelise, L., Scarpini, E., Scarlato, G., Amaducci, L., 1994. The Italian longitudinal study on aging (ILSA): design and methods. Aging Clin. Exp. Res. 6, 464–473.

McKhann, G.M., Knopman, D.S., Chertkow, H., Hyman, B.T., Jack Jr, C.R., Kawas, C.H., Klunk, W.E., Koroshetz, W.J., Manly, J.J., Mayeux, R., 2011. The diagnosis of dementia due to Alzheimer’s disease: recommendations from the National Institute on Aging‐Alzheimer’s Association workgroups on diagnostic guidelines for Alzheimer’s disease. Alzheimers Dement. 7, 263–269.

Perez-Riverol, Y., Csordas, A., Bai, J., Bernal-Llinares, M., Hewapathirana, S., Kundu, D.J., Inuganti, A., Griss, J., Mayer, G., Eisenacher, M., 2019. The PRIDE database and related tools and resources in 2019: improving support for quantification data. Nucleic Acids Res. 47, D442–D450.

Scafato, E., Gandin, C., Farchi, G., Abete, P., Baldereschi, M., Di Carlo, A., Inzitari, D., Maggi, S., Panza, F., Solfrizzi, V., 2005. Italian Project on Epidemiology of Alzheimer’s disease (I. PR. EA): study design and methodology of cross-sectional survey. Aging Clin. Exp. Res. 17, 29–34.

Tiveron, C., Fasulo, L., Capsoni, S., Malerba, F., Marinelli, S., Paoletti, F., Piccinin, S., Scardigli, R., Amato, G., Brandi, R., 2013. ProNGF\NGF imbalance triggers learning and memory deficits, neurodegeneration and spontaneous epileptic-like discharges in transgenic mice. Cell Death Differ. 20, 1017–1030.

Vanderstichele, H., Bibl, M., Engelborghs, S., Le Bastard, N., Lewczuk, P., Molinuevo, J.L., Parnetti, L., Perret‐Liaudet, A., Shaw, L.M., Teunissen, C., 2012. Standardization of preanalytical aspects of cerebrospinal fluid biomarker testing for Alzheimer’s disease diagnosis: a consensus paper from the Alzheimer’s Biomarkers Standardization Initiative. Alzheimers Dement. 8, 65–73.

Weiner, M.W., Veitch, D.P., Aisen, P.S., Beckett, L.A., Cairns, N.J., Green, R.C., Harvey, D., Jack Jr, C.R., Jagust, W., Morris, J.C., 2017. Alzheimer’s Disease Neuroimaging Initiative The Alzheimer’s Disease Neuroimaging Initiative 3: continued innovation for clinical trial improvement. Alzheimers Dement 13, 561–571.
